# Rising transphobia and disparities in drug-related harm experienced by transgender and gender-diverse people

**DOI:** 10.1186/s12954-025-01218-8

**Published:** 2025-04-18

**Authors:** Dean J. Connolly, Hari Dewan, Adam Holland

**Affiliations:** 1 National Addiction Centre, King’s College London, 4 Windsor Walk, London, SE5 8AF UK; 2 Faculty of Public Health and Policy, London School of Hygiene and Tropical Medicine, London, UK; 3 UCL Medical School, University College London, London, UK; 4 School of Psychological Sciences, University of Bristol, Bristol, UK

**Keywords:** Alcohol-related harm, Drug-related harm, Gender diverse, Harm reduction, Health inequalities, Substance-related harm, Transgender, Transphobia, United Kingdom, United States

## Abstract

**Background:**

Transgender (trans) and gender-diverse (TGD) people are a small but increasingly visible population who experience worsening marginalisation characterised by toxic political and media discourse, violent hate crimes and discriminatory laws targeting healthcare and public access. Governments in both the United Kingdom (UK) and the United States (US) have pushed anti-trans policies which threaten to further exclude TGD people. Understanding the public health impacts of transphobia is vital, especially regarding disproportionate drug-related harms.

**Main body:**

TGD people are more likely than their cisgender counterparts to experience both acute and chronic drug-related harm. This is, in part, driven by rising transphobia and perpetuated by limited access to gender-affirming and harm reduction services. Current health data systems fail to accurately capture the scale of drug-related harms faced by TGD people due to suboptimal gender measurement. Inclusive data collection and culturally competent harm reduction services are urgently needed to address these disparities. Digital interventions, such as telehealth, and peer-led support may improve the accessibility and effectiveness of care for this group.

**Conclusion:**

Evidence suggests that TGD individuals face disproportionate drug-related harm compared to cisgender people, a disparity likely to widen as government-led hostility increases in countries such as the UK and the US. Immediate action is required to ensure TGD people are fully represented in research, public health monitoring, and support services.

## Background

Transgender (trans) and gender-diverse (TGD) individuals are those whose gender identities do not correspond with their sex registered at birth. Although TGD people represent a small and minoritised group, historical erasure from representative datasets has hindered precise estimations of their population size [1]. Recent changes to the Canadian and United Kingdom (UK) censuses have begun to address this oversight, estimating that approximately 0.3% of the Canadian population and 0.5% of the UK adult population are trans or gender diverse [2, 3]. Sustained activism, popular media representation and widespread access to the internet have increased the visibility of TGD communities in many countries, which has been met with limited and decreasing tolerance [4, 5]. 

Transphobia can be characterised as a face of stigma: a complex social process, characterised by discrimination against groups who are labelled and stereotyped based on a particular behaviour or attribute [6]. Increasingly, stigma is understood as a key determinant of population health, leading to disadvantages for those in its crosshairs [7–9]. Although initially characterised as an interpersonal phenomenon [10], it is now understood to act at a structural level, conferring inequality through law, policies and social norms [11]. This theoretical approach has much in common with literature on structural violence, whereby racism, ableism, and other forms of prejudice are ingrained in policy, leading to the normalisation of disadvantage [12]. 

People who use prohibited drugs are often highly stigmatised [13, 14]. If TGD people use drugs they must then navigate intersectional stigma, with compounded negative impacts on health [15]. There has been increasing recognition that stigma is utilised as a tool of governance serving specific interests– whether or not its use is intentional or recognised by those who perpetuate it [16, 17]. Media and policy discourses frequently demonise and scapegoat migrants, welfare recipients, and people who use drugs for social problems they did not cause, deflecting attention from the structural harms of neoliberal economic policies that benefit the powerful [16, 18]. TGD people have increasingly become key targets of media and policy demonisation amid deepening socioeconomic inequality [19–21]. 

Many countries globally [22, 23], particularly the UK and United States (US), have reached a crisis state of transphobia [24], characterised by regressive public opinion [5, 25], escalating incidence of violent hate crime [26] and threatened or actual exclusion of TGD people from aspects of public life, through new legislation targeting healthcare, education, sports and bathroom access [27–30]. While a complete account is beyond the scope of this article, vitriolic political, press, and social media discourse are key precipitants, which have created an unsubstantiated moral panic [20, 23, 31, 32].

New governments in both the UK and the US have campaigned on trans exclusion. In his vow to “*end the transgender lunacy*”, Donald Trump promised voters “*…it will be a policy of the United States that there are only two genders*” [33], and UK Labour recently extended indefinitely their Conservative predecessors’ temporary criminalisation of gender-affirming medical care for young people. The latter decision was made in a consultation which included eight anti-trans organisations– among them advocates of conversion therapy– whose opinions were prioritised over the majority and clear opposition from TGD communities [34, 35]. Therefore, clinicians and policymakers must understand the public health implications of transphobia. Whilst these implications are many, in this article, we focus on the disproportionate drug-related harms experienced by TGD communities. This focus is particularly pertinent given the persistent drug-related death crises in the US, UK and elsewhere [36, 37]. 

It is important to note that whilst all drug use is associated with some level of risk, the propensity for harm varies according to the drug type and pattern of use [38, 39], with many risks exacerbated by environmental and structural conditions, including societal responses to drugs and those who use them [40]. Many policy documents unduly and disproportionately problematise all drug use among TGD people [41]. Such framing fails to account for the benefits many TGD people experience from drug use, such as pleasure, community building and identity exploration [42], as well as how the scale of risk varies between individuals and throughout the life course [43, 44]. 

### TGD people face a greater burden of drug-related harm

Collectively, relative to their cisgender (cis) peers, TGD people appear more likely to use drugs and experience a range of associated harms [45–47]. For example, TGD people are more likely to experience acute negative sequelae of drug use (e.g., blackouts and suicidality) and are at greater risk of interpersonal sexual and non-sexual violence when intoxicated [48–50]. In large-scale surveys, psychometric tests reliably find that TGD people are more likely than cis counterparts to experience drug dependence, which may negatively impact other aspects of their lives [51–53]. This is corroborated by analyses of administrative data in the US, which demonstrated a disproportionate burden of drug (and, separately, alcohol) use disorder diagnoses among TGD people [54]. This disparity is greater among transfeminine than transmasculine people highlighting that individual TGD sub-groups have unique patterns of drug use and harm [54]. 

The exact scale of this inequity is not fully understood. Issues such as sub-optimal gender measurement by clinical and public health surveillance systems and misgendering following death preclude an accurate estimation of drug-related hospitalisations, fatalities, and service use. As illicit markets for heroin and other drugs become increasingly adulterated with highly potent synthetics [37], this systemic erasure could be disastrous for TGD communities who experience concurrent exclusion from harm reduction services [55]. 

### Rising transphobia as a driver of disproportionate drug-related harm

Transphobia can be considered both a direct and indirect driver of heightened drug-related harm. Gender minority stress theories offer a framework for understanding how chronic exposure to anticipated, enacted, or internalised stigma brings about a physiological and psychological stress response that is deleterious to the health of TGD people [56–58]. In addition to contributing to physical and mental illness [59, 60], minority stress has been strongly and consistently associated with particularly harmful patterns of drug use, as people use drugs to cope despite negative effects on other areas of their lives [61–63]. 

Drug-related harm is also exacerbated indirectly through pervasive inequalities in health’s wider determinants. For example, high rates of family rejection and subsequent socioeconomic inequalities contribute to stark and widening disparities in homelessness among TGD communities [64–66]. Trans women experiencing homelessness and precarious housing report higher levels of heroin, crack and methamphetamine use relative to their housed counterparts [67]. Moreover, homelessness is strongly associated with drug-related mortality, HIV and hepatitis C transmission [68–70]. 

Transphobic policies increasingly exclude TGD people from general, gender-affirming and drug-related healthcare [28, 30, 71–73]. Delayed or inaccessible gender-affirming care prolongs gender dysphoria, a known correlate of drug use [44, 45]. Direct discrimination (including refusal of care) from individual healthcare providers, which is frequently reported by TGD people, is similarly associated with heavier drug use [71, 74, 75]. Moreover, several studies suggest that many TGD people experience othering and violence when accessing support through residential rehabilitation and from peers (e.g., in 12-step fellowships) [76–78]. In addition to escalating drug use for coping motives, discrimination in healthcare settings is associated with future healthcare avoidance [74], potentially leading to delays in seeking support for infections and other drug-related health issues [79]. 

### Recommendations for clinicians, commissioners and policymakers

Transphobia is a wicked problem and one that is likely to worsen before it improves. Therefore, the following recommendations focus on addressing drug-related harm in the context of systemic anti-trans hostility. In a recent review of highly cited quantitative, qualitative or mixed-methods alcohol research published between 2015 and 2022, measures of gender, if reported, rarely allowed the identification of TGD people [80]. Similarly, in the UK, public health surveillance reports, including for drug services and drug-related deaths, present data disaggregated by presumed (typically birth-registered) sex [81, 82], and globally gender modality and inclusive gender identity measures are lacking in most clinical records [83–85]. A shift towards inclusive measurement and reporting of both sex and gender is required when collecting data about people who use drugs. By addressing the current erasure of TGD people from these datasets, the scale and characteristics of drug-related harms they experience, both collectively and by gender identity, could be better understood and inform service planning.

Providers of harm reduction (e.g., needle and syringe programmes) and abstinence-focused services must adapt to be more inclusive of TGD people, beginning with visible allyship, cultural competence training for clinicians and robust anti-discrimination policies to address transphobia. Providers should respect and affirm the gender identity disclosed by service users through correct name and pronoun use and adopt a minority stress-informed framework when supporting TGD people [56]. Moreover, gender identity, not birth-registered sex, should inform the residential services and sex- or gender-segregated therapeutic groups and spaces to which TGD people are allocated. Community-led organisations have published comprehensive guidance on affirming service provision and inclusive data collection practices, which are generally acceptable to the wider population [86–89]. 

Historical exclusion from healthcare services and broader societal discrimination have left many TGD people with unmet health needs, including in drug-related harm reduction. Some TGD people report a preference for bespoke services and interventions that address their unique experiences and challenges [78], which is of particular importance whilst more work is needed to ensure TGD people feel welcome in gender-segregated groups and services. However, the availability of dedicated services is extremely limited in both research and practice [90–92]. This lack of access is especially pronounced outside of large, socially progressive cities, where in-person specialist services may be infeasible due to the small size and geographical dispersion of the TGD population.

Given these barriers, digital interventions present a promising alternative. Several studies have shown that some TGD people prefer digital options, citing the relative sense of safety and anonymity they provide, particularly when navigating stigmatized health issues such as drug use [78, 93]. Digital harm reduction services—such as telehealth counselling and self-help mobile apps—could be delivered at scale with relatively low resource demands, overcoming geographic and social barriers to care. When adequately resourced and supported, peer-led interventions may break down help-seeking barriers linked to anticipated discrimination, fostering trust between TGD people and the services where peer workers are embedded. Future research should prioritize testing these interventions to evaluate both their acceptability and effectiveness in reducing drug-related harm among TGD populations.

## Conclusion

All evidence suggests that TGD people experience disproportionate drug-related harm relative to cis people. This disparity appears likely to widen with increasing hostility from governments in the UK, US and elsewhere. To mitigate this harm, urgent action is required to ensure TGD people are meaningfully included in research, public health surveillance and support services.

## Data Availability

No datasets were generated or analysed during the current study.

## Abbreviations

HIV: Human immunodeficiency virus
TGD: Transgender and gender diverse
UK: United Kingdom
US: United States
